# ParaHaplo: A program package for haplotype-based whole-genome association study using parallel computing

**DOI:** 10.1186/1751-0473-4-7

**Published:** 2009-10-21

**Authors:** Kazuharu Misawa, Naoyuki Kamatani

**Affiliations:** 1 Research Program for Computational Science, Research and Development Group for Next-Generation Integrated Living Matter Simulation, Fusion of Data and Analysis Research and Development Team, RIKEN, 4-6-1 Shirokane-dai, Minato-ku, Tokyo 108-8639, Japan; 2 Laboratory for Statistical Analysis, RIKEN Center for Genomic Medicine, Tokyo, Japan

## Abstract

**Background:**

Since more than a million single-nucleotide polymorphisms (SNPs) are analyzed in any given genome-wide association study (GWAS), performing multiple comparisons can be problematic. To cope with multiple-comparison problems in GWAS, haplotype-based algorithms were developed to correct for multiple comparisons at multiple SNP loci in linkage disequilibrium. A permutation test can also control problems inherent in multiple testing; however, both the calculation of exact probability and the execution of permutation tests are time-consuming. Faster methods for calculating exact probabilities and executing permutation tests are required.

**Methods:**

We developed a set of computer programs for the parallel computation of accurate P-values in haplotype-based GWAS. Our program, ParaHaplo, is intended for workstation clusters using the Intel Message Passing Interface (MPI). We compared the performance of our algorithm to that of the regular permutation test on JPT and CHB of HapMap.

**Results:**

ParaHaplo can detect smaller differences between 2 populations than SNP-based GWAS. We also found that parallel-computing techniques made ParaHaplo 100-fold faster than a non-parallel version of the program.

**Conclusion:**

ParaHaplo is a useful tool in conducting haplotype-based GWAS. Since the data sizes of such projects continue to increase, the use of fast computations with parallel computing--such as that used in ParaHaplo--will become increasingly important. The executable binaries and program sources of ParaHaplo are available at the following address:

## Background

Recent advances in high-throughput genotyping technologies have allowed us to test allele frequency differences between case and control populations on a genome-wide scale [1]. Genome-wide association studies (GWAS) are used to compare the frequency of alleles or genotypes of a particular variant between disease cases and controls, across a given genome. A common approach is to test for differences in the allele frequencies of every single-nucleotide polymorphism (SNP) between the case and the control populations, by using the chi-square test [2-4]. The chi-square test uses the Pearson score, which increases as the difference in allele frequency between 2 populations increase. The chi-square test evaluates the Pearson score by way of the chi-square distribution.

One crucial problem in conducting SNP-based GWAS is performing corrections for multiple comparisons. A Bonferroni correction for a P-value is usually used to account for multiple testing under the assumption that all SNPs are independent. When SNP loci are in linkage disequilibrium, Bonferroni corrections are known to be too conservative and SNP-based GWAS may exclude truly significant SNPs [5,6].

To address the multiple-comparison problem in GWAS, Misawa et al. [5] have developed new algorithms to correct for multiple comparisons at multiple SNP loci in a linkage disequilibrium, by treating linked loci as one haplotype block. This approach can be referred to as haplotype-based GWAS. In the present study, a haplotype refers to a list of alleles at multiple linked polymorphic loci, while a haplotype copy denotes a list of alleles within a gamete. Misawa et al. [5] developed a method of calculating the exact probability of a type-I error of haplotype-based GWAS, under the conditions that the haplotype frequencies in the population are known and the number of haplotype copies in the sample follows a multinomial distribution. Since this algorithm calculates all possible terms, the complexity of the computational time of this exact test is O(2*n*_1_! 2 *n*_2_!), where *n*_1 _is the sample size of the case population and *n*_2 _is the sample size of the control population. When the numbers of cases and controls exceed 50, such exact probabilities cannot be calculated, since they require too much time. As an alternative method, Misawa et al. [5] developed algorithms to asymptotically calculate the type-I error rates using a Markov-chain Monte Carlo (MCMC) sampler that provides a good approximation to values calculated by the exact method. The computational complexity of the MCMC algorithm is O(*Nnm*), where *N *is the number of generations, *n *is the total sample size, *n *= *n*_1 _+ *n*_2_, and *m *is the number of loci.

The permutation test can also mitigate haplotype-based GWAS [6]. In the standard permutation test (SPT) for SNP-based GWAS, the test proceeds as follows. First, the Pearson score is calculated from the allele frequencies of the 2 populations at an SNP site; this score is the observed value of the Pearson score, S. Next, the 2 populations are pooled. The Pearson score is then calculated from the allele frequencies and recorded by randomly dividing these pooled values into two groups of size, *n*_1 _and *n*_2_. The one-sided P-value of the test is calculated as the proportion of sampled permutations where the Pearson score was greater than or equal to S. When SPT is applied to haplotype-based GWAS, haplotype copies of 2 populations are permuted, and Pearson scores are calculated for each SNP. The time complexity of the algorithm is O(*Nnm*). To search for SNPs whose P-values are lower than p, at least 1/p permutations are needed; therefore, the time complexity can be written as O(nm/p). For instance, to reach a P value of 10^-6 ^in a study that contains 1,000 cases and 1,000 controls with 10,000 loci, 10^13 ^basic computer operations are required. Obviously, scaling up to larger studies comprising 100,000 loci is completely unattainable [6]. Therefore, to obtain small P-value bounds, one must expend a great deal of computational effort. By using importance sampling, Kimmel and Shamir [6] developed the rapid association test (RAT); much less effort is needed to achieve accurate and very low P-values by RAT than by SPT. The complexity of the running time for RAT is O(*nb *+ *N nc*), where *N *is the number of permutations drawn by RAT, *b *is a predefined sampling constant, and *c *is the upper bound on the distance in SNPs between linked loci.

When the penetrance of a disease is small, a large number of SNPs from a large number of individuals from both case and control populations must be genotyped, to detect disease-associated genes [7]. There are currently more than a million SNPs for which accurate and complete genotypes have been obtained [8,9]; thus, neither the MCMC algorithm [5] nor the RAT algorithm [6] can obtain a haplotype-GWAS result.

To conduct haplotype-GWAS within a short time period, we developed ParaHaplo, a parallel-computation program that performs precisely these 2 functions for GWAS. ParaHaplo is based on data parallelism, a programming technique used to split large datasets into smaller datasets that can be run in a parallel, concurrent fashion [10]. ParaHaplo was developed on the basis of the Intel Message Passing Interface (MPI) and runs on PC clusters. ParaHaplo is a set of computer programs for SPT, RAT, MCMC, and the exact test, based on parallel computation. ParaHaplo is intended for use on workstation clusters using the Intel MPI, as well as on single-processor machines.

Using ParaHaplo, we conducted haplotype-based GWAS and SNP-based GWAS, to determine differences between Japanese in Tokyo, Japan (JPT) and Han Chinese in Beijing, China (CHB) of the HapMap dataset [11], because there are known to be small differences between JPT and CHB [9,12]. We compared the speed of calculation of our algorithm with that of the regular permutation test on chromosome 22 of JPT and CHB of HapMap.

## Implementation

### Software overview

ParaHaplo supports the HapMap data format [8], as well as those of the D-haplo DB [13] and BioBank Japan [8]. ParaHaplo requires an input file of the haplotype block boundary, as well as 2 datasets of population data. ParaHaplo can conduct either haplotype-based GWAS or SNP-based GWAS; in the case of the former, the data must be phased. ParaHaplo tests differences in allele frequency between 2 populations, e.g., a case population and a control population. ParaHaplo outputs the Pearson score for a chi-square test; a user can create ParaHaplo output by using a command-line option.

The ParaHaplo package includes both the calculation of exact probabilities and the algorithm to calculate asymptotically type-I error rates using a MCMC sampler [2]. Permutation tests SPT and RAT are also included in the ParaHaplo package. RAT is a fast algorithm for computing P-values in association studies and is based on an importance sampling developed by Kimmel and Shamir (2006) [3]. We did not incorporate the original source programs of Kimmel and Shamir (2006) into the package. The global type-I error is then obtained from the local type-I error by using a Bonferroni correction, because different haplotype blocks are considered independent of each other.

### Parallel computing using MPI methods

ParaHaplo is implemented in an MPI-C multithreaded package. The MPI package allows us to construct parallel computing programs on multiprocessors. The genome-wide polymorphism data are broken into user-defined haplotype blocks, and the MPI Bcast function is used to distribute a single set of haplotype block data into each processor. The haplotype frequency data of 1 haplotype block are analyzed by a single processor; in this step, the probability of a local type-I error is calculated, given the significance level at each SNP locus.

Once the analysis of each haplotype block is complete, the results are compiled into a single genome-wide dataset by using the MPI-Gatherv function. ParaHaplo is compatible with OpenMPI version 1.2.5, as well as MPICH version 1.2.7p1. Users can compile the source with a GCC compiler or an Intel C compiler. For single-processor machines, both the C and Java programs are also available.

## Method

### Hardware

When computational time was measured, we used a CentOS PC cluster at RIKEN comprising 1,024 nodes, each of which had a 1.6-GHz Core2duo processor. On this PC cluster, 2,048 threads can be processed in parallel. The program was compiled by an Intel C compiler. Numbers of processing unit(s) used were 1, 64, 128, 256, 512, 768, and 1536.

### Example data

As an example of GWAS, we applied ParaHaplo to compare genome-wide haplotype frequencies between JPT and CHB of HapMap [11]; the number of individuals therein were 44 and 45, respectively. In this study, we conducted 100,000 generations for RAT. Haplotype blocks were obtained as LD blocks, according to the method of Gabriel et al. [14] and using the Haploview program [15]. The entire genomes of JPT + CHB were divided into 106,149 haplotype blocks by Haploview [15].

## Results

### Haplotype-based GWAS between JPT and CHB

Table 1 shows a list of haplotype blocks whose haplotype frequencies were significantly different between JPT and CHB, as detected by ParaHaplo. ParaHaplo detected 13 haplotype blocks whose haplotype frequencies were significantly different between JPT and CHB, when the significance level was set to 0.01. In contrast, when SNP-based GWAS was conducted on the same dataset, only 2 SNPs, rs10957985 and rs10115450--which are denoted by an asterisk in Table 1--were detected as being significantly different between JPT and CHB. Since there are 1,385,520 SNPs in this dataset, SNP-based GWAS considers SNPs whose Pearson scores are greater than 33.48 as significantly different between the 2 populations at the same significance level. This result suggests that ParaHaplo, as compared to SNP-based GWAS, can detect smaller differences between 2 populations.

**Table 1:** List of haplotype blocks whose haplotype frequencies are significantly different (P < 0.01) between CHB and JPT.

**Chromosome**	**Position**	**Haplotype block**	**Number of SNPs**	**High-Score SNP**	**Score**		**Global P-value**	**Gene Name**	**Biological Function**
2	24871212	24869289-24909832	26	rs41523444	33.0		0.00774	CENPO	intron
2	205189112	205070386-205212654	69	rs12621708	33.2		0.00906	PARD3B	intron
5	18748116	18740543-18750444	5	rs11959018	32.1		0.00277		
7	134114362	134099273-134115148	10	rs3807337	29.5		0.007	CALD1	intron
8	81952476	81912934-81997651	35	rs10957985	33.9	*	0.0083		
9	103425694	103425657-103427263	5	rs10115450	43.5	*	0.00001	GRIN3A	intron
11	61080499	61049633-61102485	18	rs4939526	32.1		0.00421	SYT7	intron
11	115945221	115943365-115950641	8	rs4938285	31.3		0.00819		
12	87163821	87149707-87191176	12	rs11104775	30.4		0.00762		
13	72589237	72589149-72599947	3	rs1333099	32.1		0.00186		
15	59355415	59306354-59363106	22	rs7175875	32.7		0.00294		
18	74579176	74562645-74583266	10	rs5022079	30.0		0.00648		
22	35929011	35927436-35929568	3	rs229562	28.0		0.00711		

*Significantly different (P < 0.01) between JPT and CHB when SNP-based GWAS was used.

We found 5 genes on haplotype blocks whose haplotype frequencies were significantly different between JPT and CHB as shown in Table 1. According to OMIM [16], CENPO is a subunit of a CENPH-CENPI-associated centromeric complex that targets CENPA to centromeres and is required for proper kinetochore function and mitotic progression [17]. In Drosophila, PARD3B regulates cell polarization and is precisely regulated by 2 apically localized multiprotein signaling complexes that are tethered by Inscuteable, which regulates the apical localization [18]. CALD1 is a potential actomyosin regulatory protein found in smooth muscle and nonmuscle cells [19]. GRIN3A encodes a subunit of the N-methyl-d-aspartate (NMDA) receptors; it functions in physiological and pathological processes in the central nervous system [20]. SYT7 is a brain-specific, calcium-dependent phospholipid-binding protein that plays a role in synaptic exocytosis and neurotransmitter release [21]

### Calculation time

Speedup ratio is the ratio of the computational time of a single processor to that of multiple processors. Table 2 shows both the elapsed times and the speedups associated with the use of ParaHaplo, when chromosome 22 was analyzed. Numbers of processing unit(s) used were 1, 64, 128, 256, 512, 768, and 1536. As can be seen from table 2, calculation time decreased as the number of processors increased. When 1,536 processors were used, ParaHaplo was 100-fold faster than the non-parallel program.

**Table 2:** Elapsed times and speedups obtained with ParaHaplo on the HapMap 3 JPT data and CHB of chromosome 22

**Number of Processing Units**	**Calculation Time**			**Speed Ratio ^a^**
1	1 h	19 m	58 s	1
64		3 m	41 s	22
128		2 m	1 s	40
256		1 m	25 s	56
512			53 s	91
768			47 s	101
1536			41 s	116

^a^Ratio of Computational Time of Single Processor to Computational Time of Multiple Processors

## Discussion

We developed ParaHaplo, a set of computer programs for the parallel computation of accurate P-values in haplotype-based GWAS. ParaHaplo is intended for use in workstation clusters using the Intel MPI. By using ParaHaplo, we conducted haplotype-based GWAS as well as SNP-based GWAS, to find differences between JPT and CHB of the HapMap dataset [11].

### Differences between Japanese in Tokyo, Japan, and Han Chinese in Beijing, China

We compared the performance of our algorithm with that of the regular permutation test in comparing JPT and CHB of HapMap. By using haplotype-based GWAS, a total of 13 haplotype blocks were found to exhibit significant differences in haplotype frequency between JPT and CHB; meanwhile, by using SNP-based GWAS, only 2 SNPs were significantly different. The results suggest that ParaHaplo can detect smaller differences between 2 populations than SNP-based GWAS. Accounting for differences in substructure is necessary for improving error rates in association studies [22,23].

Natural selection is considered to be one of the causes of change in allele frequency; however, these haplotype blocks did not overlap the regions suspected of being influenced by natural selection Table 1 lists the genes on the haplotype blocks that have SNPs in which the JPT and CHP haplotype frequencies were significantly different (P < 0.01). It is unclear that the biological functions of the genes are different among JPT and CHB people.

Differences could also be caused by genetic drift [24], which brings about a change in allele frequency over time in a population, as a result of random sampling and chance. Archeological data suggest that there were probably 2 migratory waves of incoming people to Japan, both from the Asian continent. The first migration took place about 38,000-37,000 years ago, before the Pleistocene land bridges became submerged. The last ice age ended and sea levels increased around 12,000 years ago; at this point, the Japanese people became isolated from the people of mainland Asia [25]. In the 12,000 years since then, the allele frequencies of the haplotype blocks listed on Table 1 may have changed due to genetic drift. Further studies may be necessary to determine whether these differences were maintained by natural selection, genetic drift, or both.

It is generally difficult to assess how many steps are necessary for the convergence of the MCMC algorithm and RAT. In this study, we conducted 7 runs of RAT for 100,000 generations by using different sets of processing units (table 2). We found the results of these runs were essentially the same; therefore, we considered 100,000 generations to comprise a sufficiently large dataset. We recommend monitoring convergence by comparing several independent runs.

### Parallel computation of haplotype-based GWAS

The results showed that the parallel computing of ParaHaplo was 100-fold faster than non-parallel programs. In this paper, we used only 89 JPT + CHB individuals whose genotypes had been determined by the HapMap project [11]. When a single processor was used, RAT for chromosome 22 took more than 1 h; if 9,000 individuals were analyzed under the same conditions, it would take approximately 5 days. In contrast, when multiple processors were used, RAT for chromosome 22 took less than 1 min; an analysis of 9,000 individuals under the same conditions would take approximately 1 h.

There are 1,536 haplotype blocks in chromosome 22. The speedup ratio was only 116 because of variations in the LD block size. Since ParaHaplo is based on data parallelism, the computational times of each of the RAT, SPT, and MCMC methods was proportional to the number of SNPs within the LD block [5,6]; as a result, a large LD block becomes a computational bottleneck. To archive faster parallel computing of haplotype-based GWAS, further studies into more fine-grained parallelization is required.

## Conclusion

The results showed that the parallel computing of ParaHaplo was 100-fold faster than that of non-parallel programs when the number of processors is sufficient. There are more than a million SNPs for which accurate and complete genotypes have been obtained, and thousands of people are now being genotyped [8,9]. Since the data sizes of such projects continue to increase, the use of fast computations with parallel computing--such as that used in ParaHaplo--will become increasingly important.

## Availability and requirements

• **Project name**: ParaHaplo

• **Project home page**: 

• **Operating systems**: Platform independent

• **Programming language**: Java and C

• **Other requirements**: OpenMPI version 1.2.5, or MPICH version 1.2.7p1

• **License**: MIT license

• **Any restrictions to use by non-academics**: License required

## List of abbreviations used

RAT: Rapid Association Test; SPT: Standard Permutation Test; MCMC: Markov-chain Monte Carlo; JPT: Japanese Tokyo; CHB: Han Chinese Beijing.

## Competing interests

The authors declare that they have no competing interests.

## Authors' contributions

Kazuharu Misawa wrote the software and the manuscript, and Naoyuki Kamatani supervised the project. Both authors read and approved the final manuscript.
